# Assessment of Disease-Suppressive and Plant Growth-Promoting Capabilities of *Thelonectria veuillotiana*, an Endophytic Fungus Isolated from *Impatiens hawkeri*

**DOI:** 10.3390/jof12040281

**Published:** 2026-04-15

**Authors:** Huali Li, Xingyao Xiao, Mengting Luo, Jian Liu, Yajiao Sun, Mengyao Wang, Shuwen Liu, Yunqiang Ma, Hongliang Zhang, Junjia Lu

**Affiliations:** 1 College of Landscape Architecture and Horticulture Sciences, Southwest Forestry University Sciences, Kunming 650224, China; 15912938064@163.com (H.L.); 19508708561@163.com (X.X.); 13540864574@163.com (M.L.); jian927520@163.com (J.L.); 18087323192@126.com (Y.S.); 17587020327@163.com (M.W.); 15887642939@163.com (S.L.); 2 Yunnan Key Laboratory of Landscape Plant Resource Cultivation and Application, Southwest Forestry University, Kunming 650224, China; mayunqiang@swfu.edu.cn; 3 Guizhou Academy of Sciences, Guiyang 550001, China; zhl69827@sina.com

**Keywords:** *Impatiens hawkeri*, endophytic fungi, *Thelonectria veuillotiana*, biological control, growth-promoting function

## Abstract

To investigate the key role of endophytic fungi in maintaining host adaptability and overall health, endophytic fungi were isolated from healthy root, stem and leaf tissues of *Impatiens hawkeri*, and the dominant strain FG8 with growth-promoting and antagonistic functions was screened. Strain FG8 was identified as *Thelonectria veuillotiana* by morphological and molecular biological methods. It exhibited an antifungal rate of 58.57% against *Stagonosporopsis cucurbitacearum*, the pathogen causing leaf spot disease of *I. hawkeri*. The broad-spectrum antifungal activity was verified by the plate confrontation method, and FG8 showed inhibitory effects on six common pathogenic fungi, with the highest inhibition rate of 64.5% against *Apiospora intestini*. Furthermore, strain FG8 displayed remarkable growth-promoting and antagonistic characteristics: it produced indole-3-acetic acid at 12.74 μg/mL, and possessed the abilities of phosphate solubilization, potassium mobilization, nitrogen fixation and siderophore synthesis. Its antagonistic activity was mediated by β-glucanase, amylase, cellulase and pectinase. Meanwhile, FG8 significantly induced the activities of four defensive enzymes in I. hawkeri, including superoxide dismutase (SOD), peroxidase (POD), catalase (CAT) and polyphenol oxidase (PPO). Seed growth-promotion experiments demonstrated that the root length, plant height, fresh weight and dry weight of seedlings in the FG8-treated group were significantly higher than those in the control group. These results indicate that strain FG8 has both growth-promoting and biological control functions, which can provide a potential resource for the biological control of *I. hawkeri* leaf spot and the development of fungal fertilizers. Its field application effect and mechanism of action need to be further explored.

## 1. Introduction

A perennial evergreen herbaceous flower, *Impatiens hawkeri* is a member of the genus *Impatiens* in the Balsaminaceae family [1]. Due to its unique flower shape, rich and diverse flower colors, long flowering period, and strong environmental adaptability [2], it is widely used in home gardening and public landscapes and is cultivated extensively as an important ornamental flower. As a significant ornamental plant, research on the endophytic fungi of *I. hawkeri* has gradually attracted attention in recent years [3]. Endophytic fungi, as a key constituent of the plant microecosystem [4], contribute to material metabolism and energy conversion in host plants and boost host stress tolerance and disease resistance through bioactive substance production. Plant endophytic fungi are defined as fungi that establish symbiotic associations with plants without causing evident disease symptoms in their hosts [5]. Capable of infecting plant tissues, endophytic fungi affect plant growth, reproduction, and stress tolerance (biotic and abiotic) [6]. Extensive research has shown their remarkably wide distribution in the plant kingdom, identified in nearly all examined plant groups: terrestrial plants, aquatic plants, mosses, ferns, gymnosperms, and angiosperms [7].

Host plants provide the primary nutrients required for the growth and reproduction of plant endophytic fungi. They do, however, aid host plants in stress resistance and growth through the production of secondary metabolites and certain plant hormones [8,9]. For one thing, they improve host plants’ absorption of water and nutrients [9], and secrete plant growth regulators such as auxins and cytokinins to accelerate plant growth. Conversely, they enhance plants’ stress tolerance [10]; for example, they improve plants’ endurance to harsh environments such as drought, saline-alkali conditions, and heavy metals. Additionally, these organisms suppress the proliferation of pathogenic bacteria and improve plant disease resistance via the production of antibiotics, enzymes, and other such compounds. This has spurred extensive studies on plant-microbe interactions.

Research on the endophytic fungi of *I. hawkeri* is still in its infancy. Presently, systematic reports on the biological functions of endophytic fungi within *I. hawkeri* are lacking. Specifically, studies exploring the host impact of *I. hawkeri* endophytic fungi, grounded in their biocontrol and growth-promoting abilities, remain unreported. Referring to research on other plants, endophytic fungi exert beneficial effects on plant development through phosphate solubilization, potassium dissolution, nitrogen fixation, indole-3-acetic acid (IAA) production, and the synthesis of plant hormones and important metabolites [11]. Certain endophytic fungi, among them *Colletotrichum* sp., *Piriformospora* indica, and *Penicillium* sp., have received special attention for generating enzymes and metabolites that boost plant growth in extreme environments [12,13,14]. Take *Cladosporium sphaerospermum*, an endophytic fungus, which releases gibberellic acid to boost growth in rice and soybean, as an example [15]. Likewise, *Pestalotiopsis* produces pestalotin analogs—metabolites with gibberellin activity—that increase plant seed germination rates [16]. Other strains, including *Fusarium* sp., generate indole-3-acetic acid derivatives that are beneficial for plant development [17]. Furthermore, Gao et al. [18] observed that Endophytic Fungus in indica rice roots regulates host growth-related hormones and enhances nutrient uptake and translocation.

Endophytic fungi can improve soil quality by decomposing soil biomass. Some endophytic fungi are known to synthesize and secrete extracellular hydrolases [19], including pectinase, cellulase, *β*-glucanase, and protease. These hydrolases contribute to disease resistance by hydrolyzing major components of fungal cell walls, thereby inhibiting pathogen colonization and blocking nutrient acquisition from host plants [20]. Macromolecules such as polysaccharides, proteins, and organic phosphates are broken into micromolecules by them, thereby promoting host growth; these micromolecules are subsequently transported in cells to sustain metabolic processes [21]. Ayob et al. [22] isolated several filamentous fungal strains from wild *Catharanthus roseus*, which were identified as *Colletotrichum* sp., *Purpureocillium lilacinum*, *Nigrospora sphaerica*, and *Fusarium solani*. Hydrolase activity assays showed that all strains secreted cellulase universally; in addition, amylase activity was detected in *Colletotrichum* sp. and *Fusarium solani*, with the latter also producing protease. These fungi secrete extracellular enzymes that facilitate their penetration of plant cell walls and subsequent colonization. Sharma et al. [23]. evaluated the plant growth-promoting and biocontrol capabilities of endophytic fungi associated with Dioscorea, noting that 52% of isolates inhibited fungal pathogens. Some isolates also produced extracellular enzymes (e.g., amylase, lipase, protease), underscoring their potential agricultural applications.

As an important ornamental plant, *I. hawkeri* suffers from a high incidence of diseases such as leaf spot disease. Therefore, in this study, endophytic fungi were isolated from *I. hawkeri* plants with reference to previous methods, and their growth-promoting and antagonistic functions were investigated. We attempted to control plant diseases of *I. hawkeri* through biological control strategies, which can protect the environment while reducing plant diseases.

## 2. Materials and Methods

### 2.1. Materials

#### 2.1.1. Tested Endophytic Fungi of *Impatiens hawkeri*

In October 2024, *I. hawkeri* plants were acquired from Jiangsu Flower Cultivation Base Co., Ltd., (Suzhou, China), followed by six months of cultivation in the Landscape and Horticulture Laboratory of Southwest Forestry University (25°3′47″ N, 102°45′33″ E. 1948.2 m) before the experiment. The Mozhichun Seedling Business Department (Suzhou, China) provided the *I. hawkeri* seeds utilized in the present study.

#### 2.1.2. Test Strains

Several pathogenic fungi were stored in our laboratory, including strain FY9 (*Colletotrichum* sp.), *Stagonosporopsis cucurbitacearum* (causing leaf spot disease in *Impatiens hawkeri*), *Fusarium oxysporum* (inducing wilt disease in *Impatiens hawkeri*), *Pestalotiopsis* sp. (rhododendron leaf spot), *Fusarium sambucinum* (Hibiscus mutabilis leaf spot), *Botrytis cinerea* (blueberry gray mold), *Epicoccum sorghinum* (hydrangea leaf spot), and *Apiospora intestini* (ivy leaf blight). All the aforementioned strains were isolated, identified, and preserved in the Laboratory of College of Landscape Architecture and Horticulture, Southwest Forestry University.

### 2.2. Isolation of Biocontrol Fungi and Screening of Dominant Strains

Endophytic fungi isolation employed the tissue isolation method [24]. The tissue blocks of *I. hawkeri* were inoculated onto PDA plates. Sterile water from the final leaf rinse was spread onto PDA plates as a control; the absence of colony growth on the control plates indicated thorough surface disinfection. The plates were inverted and incubated in a constant-temperature incubator at 28 °C for 72 h, after which colony growth was observed. Single colonies with distinct differences in morphology, color and growth characteristics were picked for purification culture. The obtained pure strains were uniformly numbered and stored at 4 °C for subsequent use.

The plate confrontation method was employed to screen dominant strains [25]. A 7-day-old culture of *Stagonosporopsis cucurbitacearum* was punched into 6 mm discs with a fungal punch, then inoculated at the center of PDA plates via an inoculating needle. Biocontrol fungal blocks, prepared using the same method, were subsequently inoculated 2 cm from the plate center in a cross pattern. Inverted plates were incubated at 28 °C in a constant-temperature incubator for 5–7 days. In the course of incubation, antifungal zones and pathogenic fungal diameters were monitored and recorded; strains demonstrating strong pathogenic inhibition were selected as dominant.

The antifungal rate (%) = (the diameter of the control colony − the diameter of the pathogen colony)/the diameter of the control colony × 100.

### 2.3. Morphological Observation and Molecular Biological Identification of Endophytic Fungus FG8

Morphological Observation: A 6 mm diameter fungal block of endophytic strain FG8 was inoculated onto PDA medium and incubated inverted at 28 °C until the colony covered the entire plate. Characteristics such as colony color, growth status, hyphal morphology, and growth rate were observed [26]. An inoculating needle was used to pick a small quantity of hyphae, which were then mounted on a glass slide, covered with a coverslip, and observed under an optical microscope to evaluate hyphal and conidiophore morphology.

Molecular Identification: For molecular-level characterization of endophytic fungus FG8 in *I. hawkeri*, genomic DNA from the isolate was extracted via a fungal genomic DNA extraction kit according to the manufacturer’s guidelines. PCR amplification of genomic DNA was conducted with universal primers ITS1/ITS4 and gene-specific primers LSU-LR0R/LSU-LR5 [27,28], and ACT512F/ACT783R, with primer information detailed in Table 1.

PCR products were sequenced by Beijing Qingke Biotechnology Co., Ltd., (Beijing, China). Obtained sequences were submitted to NCBI, with preliminary taxonomic classification of the isolate determined via BLAST homology alignment. Multi-gene phylogenetic tree (ITS, LSU, ACT) was constructed in MEGA 11.0 using the Neighbor-Joining method, and reliability was evaluated via 1000 bootstrap replicates [29] to confirm the endophytic fungal species.

### 2.4. Determination of Growth-Promoting Characteristics of Endophytic Fungus FG8 from Impatiens hawkeri

Media for detecting nitrogen fixation, phosphate solubilization, and potassium solubilization were prepared following the method described by Zhang et al. [30]. The medium for siderophore production was obtained commercially as CAS detection medium (10.87 g dissolved in water, adjusted to a final volume of 1000 mL) [31,32]. The strain was inoculated onto nitrogen-fixing medium and incubated at 28 °C for 5 days, with continuous subculturing performed five times. The strain was considered nitrogen-fixing if it maintained growth after the third subculture; otherwise, it was deemed negative. For phosphate solubilization, potassium solubilization, and siderophore production assays, the strain was inoculated onto the respective media and incubated at 28 °C for 5 days. The formation of clear zones or halos around the colonies indicated positive growth-promoting activity, whereas their absence indicated a lack of such activity [33]. The media used are shown in Appendix A at the end of the article.

The production of IAA was assessed qualitatively and quantitatively according to the method of Imran et al. [34]. Compared to the control, a deeper color intensity corresponded to a higher level of IAA production, whereas a lighter color indicated lower production [35].

### 2.5. Determination of Antagonistic Characteristics of Endophytic Fungus FG8 from Impatiens hawkeri

Amylase, pectinase and cellulase activities were determined according to the protocol described by González et al. [36], and β-glucanase activity was measured following the method of Zhou et al. [37]. The strain was inoculated onto specific enzyme indicator media and incubated inverted at 28 °C in the dark for 5 days. The formation of clear zones or halos around colonies was used as an indicator of enzyme production. The media used are shown in Appendix A at the end of the article.

### 2.6. Determination of Broad-Spectrum Antifungal Activity of Endophytic Fungus FG8

The plate confrontation method was adopted. Plant pathogenic fungal blocks (d = 6 mm) were respectively inoculated onto PDA media using a puncher, and fungal blocks of endophytic fungus FG8 were inoculated at positions 2 cm away from the edge of the pathogenic fungal blocks. For the blank control, no FG8 fungal blocks were inoculated [38]. The experiment was conducted in triplicate, with inverted plates incubated at 28 °C in a constant-temperature incubator for 5 days. Pathogenic fungal diameters in each group were measured to determine the antifungal rate of endophytic fungus FG8.

### 2.7. Growth-Promoting Experiment of FG8 on Seed Germination and Seedling Growth of Impatiens hawkeri

Strain FG8 was inoculated into liquid Czapek medium and incubated at 28 °C with shaking at 180 r/min for 7 days. After centrifugation, the fermentation supernatant of strain FG8 was collected for subsequent use. Four hundred uniformly sized, plump *I. hawkeri* seeds with intact epidermis were selected. After disinfection, 200 seeds were immersed in the supernatant, and the remaining 200 seeds were immersed in the cell-free supernatant of PDB medium that had not been inoculated with FG8 but cultured under exactly the same conditions as FG8; both groups were shake-incubated at 37 °C and 200 r/min for 8 h. Subsequently, these treated seeds were utilized for seed germination and growth-promotion assays [39,40]. Each seed group was split into 40-seed subsets, each placed in a Petri dish lined with two layers of filter paper and moistened with a small volume of sterile water. Finally, Petri dishes were sealed with plastic wrap (pierced with several air holes) and incubated in a 25 °C constant-temperature incubator with 80% relative humidity under dark conditions. Germination was recorded on days 1, 3, 5, and 7, and seed germination rates were calculated accordingly.

Twenty-four hours after seeds in both groups (control and FG8-treated) reached peak germination, they were transplanted into nutrient soil-filled pots. Potted seedlings received regular irrigation with controlled volumes, were monitored for germination and growth, and had their root length, bud length, dry weight, and fresh weight measured and documented.

Germination rate = (Number of germinated seeds/Total number of tested seeds) × 100%

### 2.8. Endophytic Fungus FG8-Induced Resistance in Impatiens hawkeri Plants

#### Pot Culture Treatment

Pot culture treatments were conducted using the root-injury irrigation method, with 50 mL of treatment solution applied per pot. Four treatments were established: CK group was irrigated with 50 mL of sterile water, FY9 group with 50 mL of pathogenic fungus FY9 suspension (1 × 10^7^ cfu/mL), FG8 group with 50 mL of endophytic fungus FG8 suspension (1 × 10^7^ cfu/mL), and FY9 + FG8 group was first irrigated with 50 mL of pathogenic fungus FY9 suspension, followed by 50 mL of endophytic fungus FG8 suspension 24 h later. Each treatment included three replicates, with three plants per replicate, resulting in a total of 36 plants. The defensive enzyme activities of the plants were measured at 1, 3, 5, and 7 days post-treatment.

SOD enzyme activity was determined using the nitroblue tetrazolium (NBT) photoreduction method. by Taha et al. [41]. POD activity was determined using the guaiacol colorimetric method, and CAT activity was measured by the ultraviolet spectrophotometric method based on hydrogen peroxide decomposition, with reference to the method described by Li et al. [42]. PPO activity was determined using the catechol colorimetric method, with the procedure adapted from Shen et al. [43].

### 2.9. Physiological Characterization of Endophytic Fungus FG8

#### 2.9.1. Growth of FG8 on Different Carbon Sources

Using Czapek Medium as the base, glucose was replaced with equal amounts of various carbon sources (soluble sugar, mannitol, maltose, lactose, sucrose, etc.). Three replicates were established for each treatment [44]. The medium was inoculated with activated FG8 fungal blocks, incubated at 28 °C for 5 days, and colony diameters were subsequently assessed using the cross-measurement method. Each carbon source was added at a concentration of 20 g/L to replace glucose.

#### 2.9.2. Growth of FG8 on Different Nitrogen Sources

Czapek Medium served as the base, with various nitrogen sources (peptone, sodium nitrate, yeast extract powder, ammonium sulfate, beef extract, ammonium chloride, among others) added separately. Each treatment included three replicates. Following inoculation of activated FG8 fungal blocks onto the medium, incubation at 28 °C for 5 days was performed, with colony diameters quantified via the cross-streak method. Each nitrogen source was added at a final concentration of 2 g/L.

#### 2.9.3. Growth of FG8 on Different Media

FG8 was inoculated onto PDA, PPDA, SDAY, Czapek, cornmeal, and water agar media. Triplicate replicates were set for each treatment. Inoculated and activated FG8 fungal blocks were incubated at 28 °C for 5 days, subsequent to which colony diameters were measured via the cross method.

#### 2.9.4. Growth of FG8 at Different Temperatures

Inoculation of activated FG8 fungal blocks onto PDA medium was followed by dark incubation at 5 °C, 10 °C, 15 °C, 20 °C, 25 °C, 30 °C, 35 °C, and 40 °C. Each treatment was performed in triplicate, and colony diameters were quantified via the cross-measurement method after 5 days of incubation.

#### 2.9.5. Growth of FG8 at Different pH Levels

Prepare PDA medium. According to the target pH gradients (5, 6, 7, 8, 9, 10, 11), adjust the pH using sterile 1 mol/L HCl solution and 1 mol/L NaOH solution respectively: place the medium to be adjusted on a magnetic stirrer and stir slowly, add the adjusting reagent drop by drop, pause stirring after every 3–5 drops, measure the current pH value with a calibrated pH meter, and repeat the operation until the target pH value is reached. After pouring the medium into plates, inoculate the activated FG8 fungal blocks, incubate at 28 °C for 5 days, and measure the colony diameter using the cross method.

### 2.10. Data Analysis

Excel 2016 and SPSS 22.0 facilitated data analysis. Statistical significance of samples was assessed via one-way ANOVA (*p* < 0.05), followed by Duncan’s multi-range test. Adobe Photoshop 2023 and Adobe Illustrator 2020 supported data visualization.

## 3. Results

### 3.1. Isolation and Screening of Endophytic Fungi from Impatiens hawkeri

**Figure 1:**
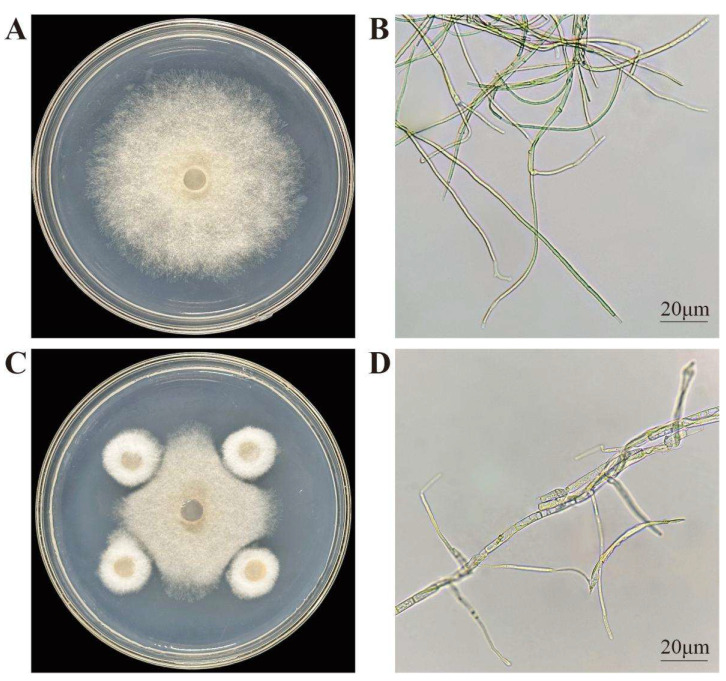
Effect of FG8 on the mycelium of *S. cucurbitacearum*. (**A**) Control colony; (**B**) Control hyphae; (**C**) Inhibition of the FG8 strain on *S. cucurbitacearum*; (**D**) Mycelia after inoculation with the FG8 strain. *S. cucurbitacearum* and FG8 were incubated at 28 °C in the dark for 5 d.

From *I. hawkeri*, 21 endophytic fungal strains were isolated. Plate-based antimicrobial screening against its leaf spot pathogen was performed on these strains to identify those with strong inhibitory activity. Root-isolated strains were designated “FG,” stem-derived ones “FJ,” and leaf-isolated ones “FY”. Among them, the endophytic fungus to be tested with the best inhibitory effect was FG8, and the antifungal effect of FG8 is shown in Figure 1. By observing the pathogenic fungal hyphae of the two treatment groups under an optical microscope, in the control group, mycelia of the pathogenic fungus *S. cucurbitacearum* exhibited a smooth, slender morphology with uniform thickness and no excessive branching; in contrast, FG8-inhibited *S. cucurbitacearum* mycelia displayed characteristics including thickening, swelling, distortion, and increased branching. This indicates that the biocontrol fungus FG8 has a destructive effect on the mycelia of the pathogenic fungus.

### 3.2. Morphological and Molecular Biological Identification of Endophytic Fungus FG8

#### 3.2.1. Morphological Identification

**Figure 2:**
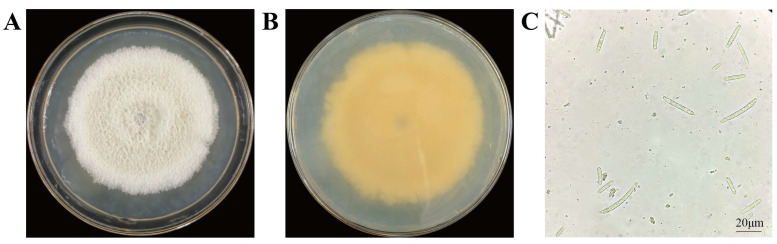
Colony and spore morphology of FG8. FG8 was incubated upside down at 28 °C in the dark for 7 d. (**A**,**B**) Obverse and reverse of FG8 colony; (**C**) Conidia of FG8 under different fields of view.

The colony of endophytic fungus FG8 presents as white fluffy villi with radial growth from the center outward; its mycelia are relatively densely arranged, and the surface is moist with an overall opaque appearance. The colony binds tightly to the medium and has strong adhesiveness, making it difficult to completely pick up the mycelial tissue with a conventional inoculating needle. Under an optical microscope, the conidia are single-celled, green, cylindrical or clavate (slightly curved), and distributed irregularly and scatteredly. Based on the relevant literature [45], strain FG8 was preliminarily identified as *Thelonectria veuillotiana*. These characteristics are illustrated in Figure 2.

#### 3.2.2. Molecular Biological Identification

**Figure 3:**
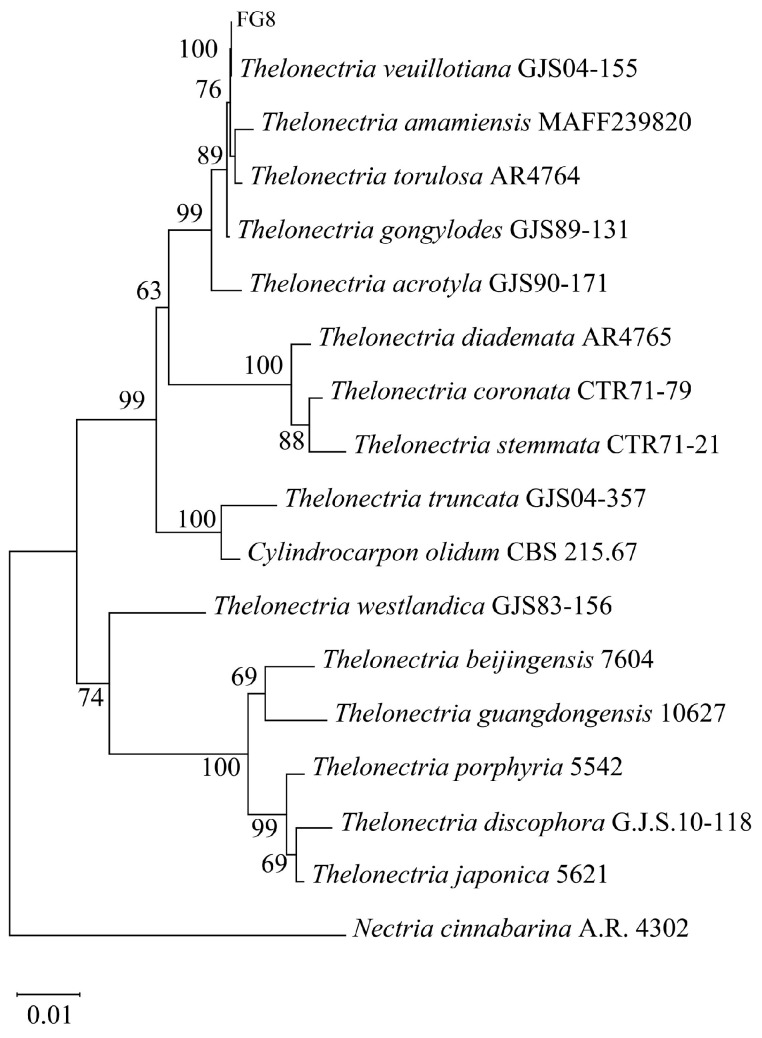
Neighbor-joining multigene phylogenetic tree of the fungus FG8 was constructed by MEGA 11.0.

DNA was extracted from strain FG8 for sequencing, and a phylogenetic tree was constructed as shown in Figure 3. Sequence alignment results revealed a 100% bootstrap support value between FG8 and *Thelonectria veuillotiana*, and combined with morphological traits, strain FG8 was identified as *T. veuillotiana*.

### 3.3. Determination of Growth-Promoting and Antagonistic Abilities of Endophytic Fungi from Impatiens hawkeri

#### 3.3.1. Determination of Growth-Promoting and Enzyme-Producing Abilities of Endophytic Fungi from *Impatiens hawkeri*

**Figure 4:**
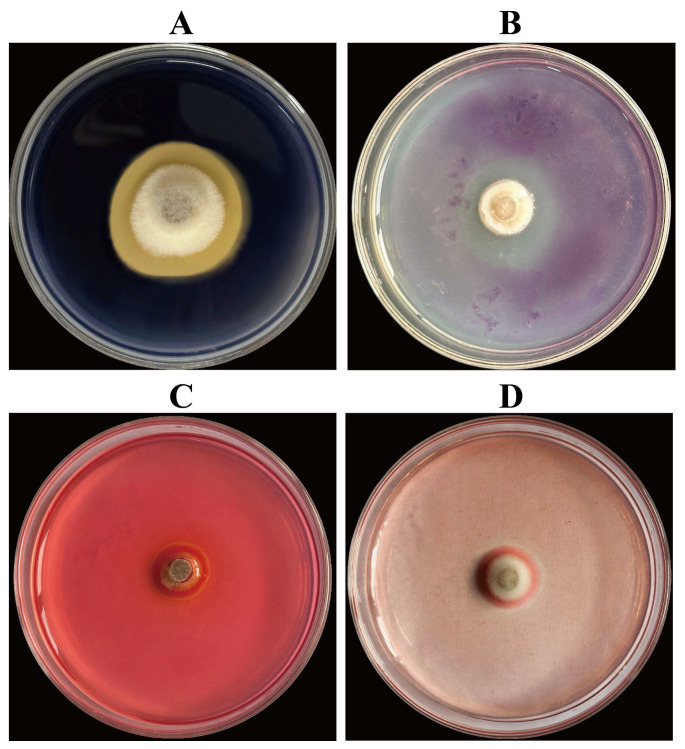
Enzyme production status of five strains of antagonistic fungi. Enzyme Production of Cell Wall Hydrolases by Endophytic Fungus Strain FG8. (**A**) Amylase-producing; (**B**) Pectinase-producing; (**C**) Cellulase-producing; (**D**) *β*-glucanase-producing. FG8 was cultured in inversion on various media at a constant temperature of 28 °C in the dark for 3 d.

**Figure 5:**
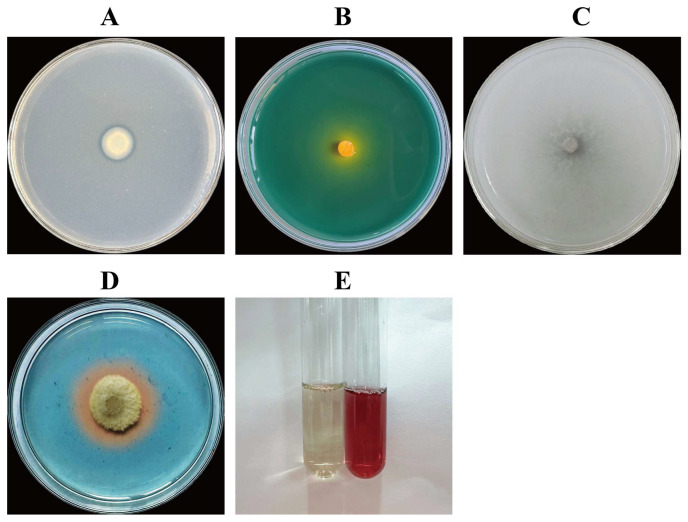
Detection of Growth-Promoting Function of Endophytic Fungus Strain FG8. (**A**) Phosphate-solubilizing ability; (**B**) Potassium-solubilizing ability; (**C**) Nitrogen-fixing ability; (**D**) siderophore-producing ability; (**E**) IAA-producing abilities. (**A**–**D**) FG8 cultured upside down on various media at 30 °C in the dark for 3 d.

Strain FG8 exhibits phosphate and potassium solubilization, nitrogen fixation, siderophore biosynthesis, and production of pectinase, amylase, cellulase, and *β*-glucanase, as presented in Table 2, Figure 4 and Figure 5.

#### 3.3.2. Determination of Antifungal Activity of Endophytic Fungi from *Impatiens hawkeri*

**Figure 6:**
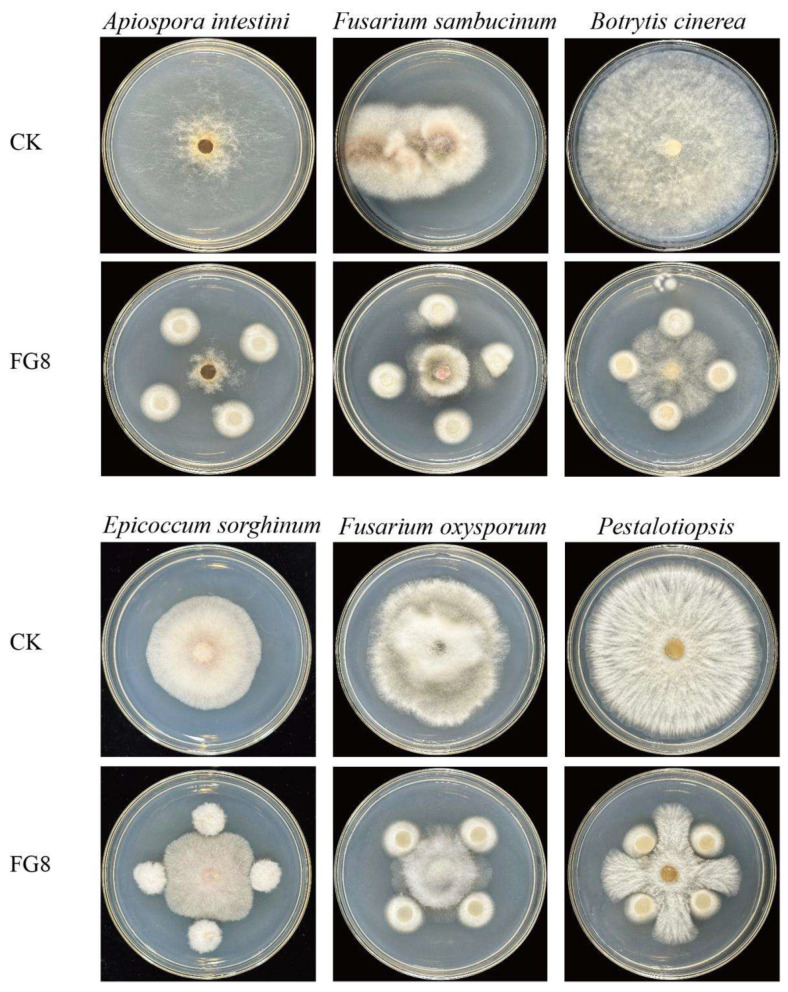
The impact of antagonistic fungus FG8 on different pathogenic fungi. FG8 and various pathogenic fungi were cultured in inversion at 28 °C in the dark for 5 d.

Strain FG8 can inhibit the growth of 6 pathogenic fungi: *Fusarium oxysporum*, *Pestalotiopsis* sp., *Fusarium sambucinum*, *Apiospora intestini*, *Epicoccum sorghinum*, and *Botrytis cinerea*, with the highest inhibition rate of 64.5% against *Apiospora intestini*. As presented in Table 3, Figure 6. It restricts the outward expansion of their colonies and forms distinct antifungal zones, suggesting that it may synthesize antifungal metabolites.

### 3.4. Growth-Promoting Effect of FG8 on Seeds and Seedlings of Impatiens hawkeri

Within 36 h of the seed germination assay, both control and FG8-treated *I. hawkeri* seeds began to germinate. At 72 h, the control group achieved maximum germination. Conversely, FG8 treatment accelerated germination, resulting in a higher germination rate, earlier maximum germination (48 h), and significantly longer roots compared to the control.

**Figure 7:**
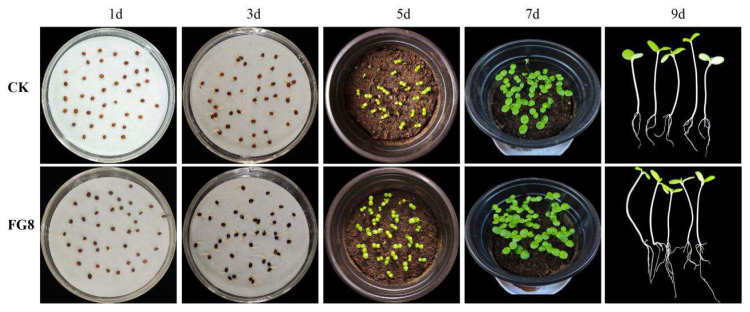
Promoting effect of FG8 on the seed germination of *Impatiens hawkeri*.

Some control seedlings had broken through the soil five days after transplantation into flowerpots, exposing green new leaves and stems, whereas a few stayed underground, with growth becoming uneven as a result. By contrast, nearly all seedlings receiving FG8 treatment had emerged, with visible green stems and new leaves; only a few had not developed cotyledons, and sprouts showed relatively uniform growth. By day 7, all control seedlings had emerged, but growth inconsistency became more intense. The FG8-treated group showed more vigorous growth, with uniform new leaf sizes and plant heights across seedlings. When seedlings from each group were measured randomly, significant differences in root length, bud length, and plant height were found (*p* < 0.05). In the FG8-treated group, seedling height reached 16.62 cm, germination rate 97.5%, fresh weight 10.15 g, and dry weight 0.39 g, whereas the control group had a seedling height of 12.98 cm, germination rate of 90.63%, fresh weight of 7.47 g, and dry weight of 0.2954 g; other parameters including root length and stem length were also greater in the treated group. From data including germination rate, seedling height, fresh weight, and dry weight, it can be seen that the strain FG8 has a good growth-promoting effect on the seeds and seedlings of *I. hawkeri*. As presented in Table 4, Figure 7.

### 3.5. Induction of Disease Resistance Activity in Impatiens hawkeri by Strain FG8

#### 3.5.1. Dynamic Changes in SOD Activity

**Figure 8:**
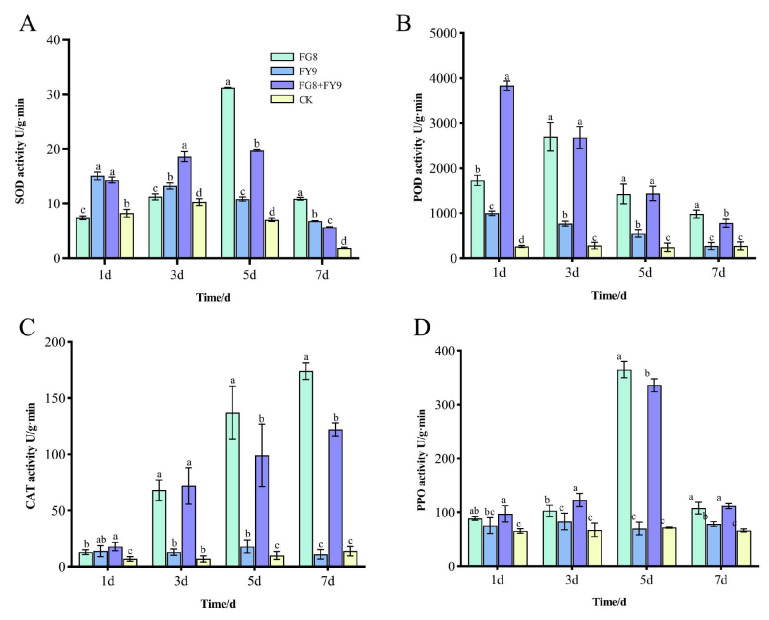
(**A**) SOD enzyme activity. (**B**) POD enzyme activity. (**C**) CAT enzyme activity. (**D**) PPO enzyme activity. Different letters above bars indicate statistically significant differences between groups at the *p* < 0.05 level.

As a key enzyme responsible for scavenging superoxide anion radicals and alleviating oxidative damage, SOD activity exhibited distinct time-dependent and treatment-specific patterns. At 1 day post-treatment, stress induced by the pathogenic fungus FY9 rapidly upregulated SOD activity. No significant difference was observed in SOD activity between the combined treatment group (FG8 + FY9) and the FY9 single-treatment group, while both groups showed significantly higher SOD activity than the FG8 single-treatment group and the blank control (CK). These results indicated that pathogenic stress could rapidly activate the antioxidant response in plants. At 3 days post-treatment, the SOD activity in the combined treatment group reached 18.62 U/g·min, which was significantly higher than that in the FY9 single-treatment group (13.27 U/g·min), and also higher than those in the FG8 single-treatment group and the CK group. This finding demonstrated that the synergistic effect of the endophytic fungus FG8 and the pathogen further enhanced the antioxidant defense response of the host plants. At 5 days post-treatment, the SOD activity in the FG8 single-treatment group peaked at 31.20 U/g·min, which might be attributed to the gradual stabilization of the synergistic interaction between the endophytic fungus and the host plant (Figure 8A).

In addition, the SOD activity in the FG8 + FY9 combined treatment group remained higher than that in the FY9 single-treatment group, confirming the persistent synergistic effect of FG8. At 7 days post-treatment, the SOD activity showed an overall decline across all treatment groups; the activity in the combined treatment group was slightly lower than that in the FY9 single-treatment group but still higher than that in the CK group, suggesting that the protective effect of FG8 attenuated gradually over time. Collectively, these results indicated that the endophytic fungus FG8 could effectively synergize to enhance SOD activity during the early stage of pathogenic fungal stress (1–3 days post-treatment), thereby strengthening the plant’s capacity to resist oxidative damage.

#### 3.5.2. Dynamic Changes in POD Activity

Peroxidase is involved in the oxidation of phenolic substances and the lignification of cell walls, serving as a crucial indicator for plant disease resistance. Its activity exhibits the most sensitive response to various treatments. At 1 day post-treatment, the POD activity in the combined treatment group reached 3830 U/g·min, which was significantly higher than that in the other groups. The FG8 group (1730 U/g·min) followed, while the FY9 group (1000 U/g·min) and the control group (CK, 260 U/g·min) showed relatively low activity. These results indicated that the synergistic effect of FG8 and FY9 could rapidly induce a substantial increase in POD activity, potentially enhancing the early defense capability of plants by accelerating cell wall lignification (Figure 8B).

On day 3 after treatment, there was no significant difference in POD activity between the FG8 group and the combined treatment group, and both groups maintained significantly higher POD activity than the FY9 group and the CK group, indicating that FG8 effectively maintained a high level of POD activity. On days 5 and 7 after treatment, although POD activity in the combined treatment group declined to some extent, it remained at a relatively high level comparable to that in the FG8 group and was still significantly higher than that in the FY9 group and the CK group. In contrast, POD activity in the FY9 group remained consistently low during this period. It is speculated that the pathogenic fungus may inhibit POD synthesis, whereas FG8 may alleviate this inhibition and delay the rapid decline in POD activity.

#### 3.5.3. Dynamic Changes in CAT Activity

Catalase is primarily responsible for decomposing hydrogen peroxide (H_2_O_2_), thereby preventing oxidative stress-induced damage to plants. The variation in its activity exhibited an overall trend of FG8 + FY9 group ≈ FG8 group > FY9 group > CK group.

At 1 day post-treatment, there was no significant difference in CAT activity between the combined treatment group and the FY9 group, while both groups showed higher activity levels than the FG8 group and the CK group. This indicated that pathogenic stress rapidly activated the defensive function of CAT. On the 3rd day post-treatment, the CAT activity in the combined treatment group reached 72 U/g·min, which was comparable to that in the FG8 group (68 U/g·min) and significantly higher than that in the FY9 group (13 U/g·min) and the CK group (7 U/g·min). These results reflected the capacity of FG8 to enhance H_2_O_2_ decomposition. At 5 and 7 days post-treatment, the FG8 group consistently maintained the highest CAT activity, followed closely by the combined treatment group. In contrast, the FY9 group sustained relatively low activity levels, with the CK group showing the lowest activity. These findings suggested that individual pathogenic stress could inhibit CAT activity, whereas the endophytic fungus FG8 could effectively alleviate such inhibition, continuously enhance the plant’s ability to scavenge H_2_O_2_, and reduce oxidative damage (Figure 8C).

#### 3.5.4. Dynamic Changes in PPO Activity

Polyphenol oxidase can catalyze the oxidation of phenolic substances to quinones, thereby inhibiting the growth of pathogenic fungus. Its activity exhibited the most stable response to the combined treatment.

At 1 day post-treatment, the PPO activity of the combined treatment group (97.2 U/g·min) was significantly higher than that of the other groups. There was no significant difference in PPO activity between the FG8 group (89.2 U/g·min) and the FY9 group (75.6 U/g·min), while the water control group (65.5 U/g·min) showed the lowest activity. These results indicated that the synergistic effect of FG8 and FY9 could rapidly enhance the phenolic oxidation defense of plants.

On the 3rd day post-treatment, the PPO activity of the combined treatment group further increased (123 U/g·min), which was significantly higher than that of the FG8 group, FY9 group and CK group. This demonstrated that FG8 continuously promoted the synthesis of quinones and strengthened the inhibitory effect on pathogenic fungus.

On the 5th day post-treatment, the PPO activity of the CK group reached a peak (365 U/g·min), while that of the combined treatment group was 336 U/g·min, significantly higher than that of the FY9 group and CK group (Figure 8D).

On the 7th day post-treatment, there was no significant difference in PPO activity between the FG8 group and the combined treatment group, and both groups maintained higher activity levels than the FY9 group and CK group. These findings suggested that the synergistic defensive effect of FG8 persisted throughout the entire experimental period, effectively maintaining the disease resistance of plants.

In summary, the endophytic fungus FG8 exerted a significant enhancing effect on the resistance of Impatiens hawkeri to the stress of pathogenic fungus FY9, and its core mechanism was achieved by increasing the activity of key defensive enzymes. In the early stage of stress (1–3 days), FG8 and the pathogenic fungus synergistically induced a rapid increase in the activities of SOD, POD and PPO, thereby enhancing the plant’s antioxidant damage capacity and early defense capability. Throughout the entire experimental period, FG8 effectively alleviated the inhibitory effect of the pathogenic fungus on CAT activity, reduced the accumulation of H_2_O_2_, and simultaneously maintained a high level of PPO activity, inhibiting the growth of pathogenic fungus through phenolic oxidation. Different enzymes exhibited distinct responses to the treatments: POD and PPO were the most sensitive to the combined treatment, reflecting the rapid synergistic effect of early defense; SOD activity showed the most significant advantage in the middle stage, and its effect weakened in the later stage. When treated with the pathogenic fungus FY9 alone, the activities of the four defensive enzymes were significantly lower than those in the combined treatment group and the CK group at most time points, indicating that individual pathogenic stress could weaken the plant’s defense capability. Meanwhile, the activity of the water control group remained the lowest throughout the experiment, which further verified the protective effect of the endophytic fungus FG8.

### 3.6. Analysis of Biological Characteristics of Endophytic Fungus FG8

**Figure 9:**
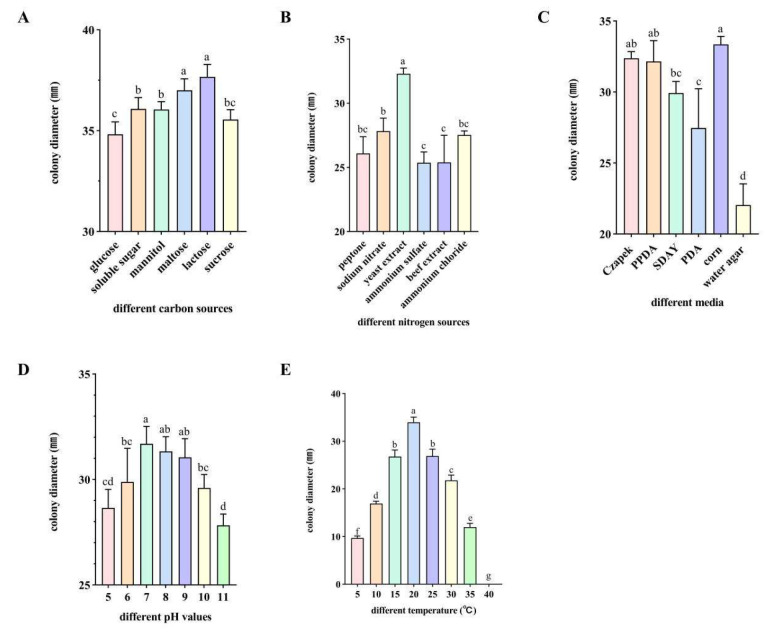
(**A**) Different carbon sources. (**B**) Different nitrogen sources. (**C**) Different culture media. (**D**) Different pH values. (**E**) Different temperatures. Different letters above bars indicate statistically significant differences between groups at the *p* < 0.05 level.

By analyzing the impacts of various environmental factors (carbon sources, nitrogen sources, culture media, temperature, pH) on strain FG8, this study explored the relationship between its occurrence patterns and environmental conditions. This work lays a theoretical foundation for exploring the growth conditions of endophytic fungi from *I. hawkeri*. Significant difference analysis was performed on data from different treatments using GraphPad Prism 9, following the one-way analysis of variance (ANOVA) model. As shown in Figure 9.

#### 3.6.1. Effect of Different Carbon Sources on the Growth Rate of Strain FG8

The pathogen grew across various carbon sources, with substantial differences in growth rates. Among the six tested carbon sources, strain FG8 displayed significant variations in colony growth. Following 7 days of culture, faster growth was observed in lactose- and sucrose-containing media, with colony diameters reaching 37.67 mm and 37.00 mm, respectively; growth was slowest in glucose-based medium, where the colony diameter measured 34.82 mm.

#### 3.6.2. Effect of Different Nitrogen Sources on the Growth Rate of Strain FG8

Under different nitrogen source conditions, strain FG8 could grow, and its growth status varied significantly. Among the 6 tested nitrogen sources, strain FG8 showed a better utilization effect on yeast extract. After 7 days of incubation, the colony had a diameter of 32.31 mm. The weakest utilization by the strain was observed for ammonium sulfate and beef extract, with colony diameters of 25.37 mm and 25.39 mm corresponding to these.

#### 3.6.3. Effect of Different Culture Media on the Growth Rate of Strain FG8

Across diverse culture media, FG8 exhibited marked variations in growth rate: it grew fastest on corn agar medium (average colony diameter reaching 33.35 mm) and slowest on water agar medium (colony diameter 22.04 mm).

#### 3.6.4. Effect of pH on the Growth Rate of Strain FG8

After culturing strain FG8 on media with pH ranging from 5 to 11 at 25 °C for 7 days, observation showed that the growth rate was slightly higher at pH 7, with a colony diameter of 31.70 mm, while the growth was the slowest at pH 11, with a colony diameter of 27.83 mm.

#### 3.6.5. Effect of Temperature on the Growth Rate of Strain FG8

Temperature exerted a significant impact on the pathogen’s growth rate. Strain FG8 exhibited a growth pattern of initial increase followed by decline with temperature fluctuations, viable between 5 and 35 °C but incapable of mycelial growth at 40 °C. Mycelia grew sparsely at 5 °C, slowly at 10–15 °C and 25–30 °C, and most rapidly at 20 °C, reaching an average colony diameter of 33.98 mm after 7 days.

## 4. Discussion

*Thelonectria veuillotiana* was initially assigned to the genus *Nectria* (family Nectriaceae). However, taxonomic studies in 2011, based on multi-gene phylogenetic analyses and other complementary approaches, reclassified this fungus into the newly established genus *Thelonectria*, with its official scientific name revised to *T. veuillotiana* [46]. In addition, it has several synonyms including *Dialonectria veuillotiana* and *Cucurbitaria veuillotiana*. The genus Thelonectria exhibits diverse ecological habits and rich species diversity, thus holding significant economic importance. Most species function as saprophytes or plant pathogens, while some act as mycoparasites or entomophagous fungi; certain taxa have also been reported as opportunistic pathogens in humans [47]. However, the strain of *T. veuillotiana* isolated from *I. hawkeri* in this study was identified as a plant endophytic fungus, which is not only non-pathogenic, but also exhibits inhibitory effects against pathogenic fungi and exerts alleviative effects on plant diseases.

A strain of the endophytic fungus *T. veuillotiana* was isolated from *Oreorchis patens*, a rare native orchid species in South Korea, in a previous study. The study further noted that although this strain is widely distributed on the bark of trees and shrubs and can occasionally act as a phytopathogen, it did not exhibit pathogenicity toward its host when isolated from the roots of *Oreorchis patens*, instead existing as a symbiotic endophytic fungus [48]. Similarly, strain FG8 isolated in this study not only fails to induce diseases in *I. hawkeri*, but also exerts favorable inhibitory effects against the phytopathogenic fungus causing *I. hawkeri* leaf spot as well as six other categories of phytopathogenic fungi, and can induce the activities of plant defense enzymes such as superoxide dismutase (SOD). In addition, functional assays for plant growth promotion and seed germination tests have verified that strain FG8 exhibits a prominent growth-promoting effect on Impatiens hawkeri seeds. This further validates that some strains generally regarded as phytopathogens are capable of colonizing plants without inciting diseases, while also enhancing plant disease resistance and exerting plant growth-promoting effects.

The key functional basis for the biocontrol efficacy of FG8 lies in its ability to produce a variety of hydrolytic enzymes, including amylase, pectinase, cellulase, and *β*-glucanase. Among these, *β*-glucanase is considered capable of degrading the cell walls of phytopathogens, thereby weakening their ability to colonize host plants and cause disease [49]. Similar enzyme-mediated antagonistic effects have also been reported in endophytic Trichoderma species, in which cellulase and *β*-glucanase contribute to the suppression of fungal pathogens such as *Fusarium* and *Alternaria* [50].The capacity of FG8 to produce multiple cell wall-degrading enzymes and defense-related enzymes further supports the hypothesis that it is a highly efficient biocontrol fungus; it may not only directly inhibit pathogens, but also induce defense responses in the host plant [51].

In terms of plant growth promotion, FG8 demonstrates a suite of traits that collectively enhance *I. hawkeri* performance. Its capacity to solubilize phosphate, mobilize potassium, fix nitrogen, produce siderophores, and synthesize IAA reflects the functional diversity of beneficial rhizosphere microorganisms emphasized in recent reviews [52,53]. Phosphate and potassium solubilization, for instance, increases the bioavailability of these essential macronutrients, addressing a common limitation in agricultural soils [54]. Nitrogen fixation further supplements soil fertility, while siderophores enhance iron uptake—a critical micronutrient for chlorophyll synthesis and enzyme function [55]. Additionally, IAA biosynthesis directly drives cell elongation and division, as reflected in FG8’s enhancement of *I. hawkeri* seed germination and seedling growth—root length, plant height, and fresh/dry weight were significantly higher than controls. This concurs with Wenkun et al., who observed that endophytic IAA producers substantially improved growth parameters in horticultural crops [56].The growth-promoting functions of endophytic fungi (including phosphate solubilization, potassium solubilization, nitrogen fixation, siderophore production, and indole-3-acetic acid (IAA) synthesis) are primarily achieved through the secretion of specific metabolites to mediate substrate transformation and nutrient release. The type, concentration, and synergistic effects of these metabolites directly determine the functional intensity, which is also closely associated with fungal strain species and environmental conditions.

Compared with the maximum phosphate-solubilizing capacity of *Aspergillus flavus* JKJ7 (762.32 mg/L) reported by Moropana et al., the phosphate-solubilizing ability of FG8 shown in Table 2 was only 24.76 μg/mL. Nevertheless, the IAA-producing and siderophore-producing capacities of FG8 were similar to those determined by Moropana et al. [57]. With regard to the phosphate-solubilizing ability, comprehensive analysis suggests that the discrepancy may arise from differences in the liquid medium used, measurement time points, and species or strain variations. Moreover, considering the growth-promoting effect of FG8 on seed germination, its plant growth-promoting function cannot be negated. Further research should be conducted in future studies to explore the dynamic changes in its phosphate-solubilizing capacity and plant metabolic responses across different time gradients, so as to elucidate its functional traits in greater detail.

Endophytic fungi do not directly “synthesize” nutrients; instead, they rely on their metabolites to convert insoluble phosphorus and mineral potassium in the soil into forms that are available for plant uptake. The well-characterized biological traits of FG8, including its optimal carbon and nitrogen sources, preferred medium, temperature, and pH, are pivotal for its practical application. As noted by Kim et al., standardized growth conditions are indispensable for scaling endophyte production to ensure consistent metabolite yields and functional efficacy [58]. FG8’s well-defined growth requirements streamline industrial cultivation, a significant advantage over poorly characterized strains that show variable performance under diverse conditions.

With the increasing demand for biological control, the disease resistance and growth-promoting effects of FG8 on plants warrant further in-depth investigation. Field trials are necessary to validate FG8’s efficacy under complex environmental conditions, as greenhouse performance often differs from real-world agricultural settings [59]. Moreover, investigating the persistence of FG8 in soil and plant tissues, alongside its potential interactions with other soil microorganisms, will be key to elucidating its ecological role [60]. Future studies should further explore the molecular mechanisms governing FG8’s dual functions, including gene expression patterns under pathogen stress or nutrient restriction, to optimize its application.

## 5. Conclusions

In this study, an endophytic fungus *Thelonectria veuillotiana,* with both plant growth-promoting and biocontrol properties, was isolated from *Impatiens hawkeri*. Its growth-promoting function is mainly achieved by transforming nutrients for plant uptake. Its biocontrol effect is exerted through two major pathways: on the one hand, it secretes cell wall-degrading enzymes to directly suppress the proliferation of pathogenic fungi; on the other hand, it upregulates the activities of host defense enzymes to enhance intrinsic disease resistance, thereby providing a dual protective effect against pathogens. In addition, this strain is easy to culture under laboratory conditions. Collectively, endophytic fungus FG8 is a high-quality microbial resource with dual functions of plant disease biocontrol and growth promotion. It presents important application value in the green cultivation, leaf spot management, and microbial fertilizer development of *I. hawkeri*, and also provides theoretical support for the exploration and utilization of endophytic fungal resources in ornamental flowers. This study was only conducted under laboratory and pot conditions. Future research may further carry out field validation, dissect the strain–host interaction mechanism, and optimize fermentation processes to accelerate its popularization and application in large-scale cultivation.

## Figures and Tables

**Table 1:** Primer sequences and reaction programs for FG8 sequencing.

Primers		Primer Sequences	PCR Program
ITS	ITS1	TCCGTAGGTGAACCTGCGG	pre-denaturation at 94 °C for 5 min, 35 cycles of 94 °C denaturation (30 s), 55 °C annealing (30 s), and 72 °C extension (1 min), and a final 72 °C extension for 10 min.
	ITS4	TCCTCCGCTTATTGATATGC	
LSU	LR0R	GTACCCGCTGAACTTAAGC	pre-denaturation at 94 °C for 5 min, 35 cycles of 94 °C denaturation (45 s), 52 °C annealing (30 s), and 72 °C extension (1 min), and a final 72 °C extension for 10 min.
	LR5	ATCCTGAGGGAAACTTCG	
ACT	ACT512F	ATGTGCAAGGCCGGTTTCGC	pre-denaturation at 94 °C for 5 min, 35 cycles of 94 °C denaturation (30 s), 58 °C annealing (30 s), and 72 °C extension (45 s), and a final 72 °C extension for 10 min.
	ACT783R	TACGAGTCCTTCTGGCCCAT	

**Table 2:** Analysis of Growth-Promoting and Enzyme-Producing Capabilities of Endophytic Fungus FG8.

Index	Quantitative	Index	Quantitative
Soluble phosphorus experiment	24.76 μg/mL	Starch hydrolysis	198.24 U/mL·min
Potassium dissolving experiment	6.51 μg/mL	Cellulase production experiment	160.77 U/mL·min
IAA production experiment	12.74 μg/mL	Produce pectinase	48.91 U/mL·min
Experiment of producing siderophore	20.02%	Produce *β*-glucanase	74.66 U/mL·min

**Table 3:** Effects of Antagonistic Fungus FG8 on Different Pathogenic Fungi.

Pathogenic Fungi	Inhibition (%)	Pathogenic Fungi	Inhibition (%)
*Apiospora intestini*	64.50 ± 2.54 a	*Fusarium sambucinum*	54.77 ± 3.06 b
*Botrytis cinerea*	46.37 ± 3.77 c	*Epicoccum sorghinum*	44.73 ± 3.25 c
*Fusarium oxysporum*	48.08 ± 1.74 c	*Pestalotiopsis*	32.03 ± 0.58 d

Different letters after data indicate significant differences (*p* < 0.05).

**Table 4:** The effects of FG8 on the seed germination and seedling growth of *Impatiens hawkeri*.

Treatment	Plant Height (cm)	Root Length (cm)	Sending Length (cm)	Germination Rate (%)	Fresh Weight (g)	Dry Weight (g)
Control	12.98 b	5.21 b	6.61 b	90.63 b	7.47 b	0.2954 b
FG8	16.62 a	7.40 a	8.07 a	97.5 a	10.15 a	0.3900 a

Different letters after data indicate significant differences (*p* < 0.05).

## Data Availability

The original contributions presented in this study are included in the article. Reagents, microbial materials, and datasets used, created, and analyzed in the study are available upon request to the corresponding author. The genomic sequencing data mentioned in this study are stored in the Sequence Read Archive (SRA) database of the National Center for Biotechnology Information (NCBI) and can be accessed via the NCBI database. The source data are presented in the form of additional source datasets. All sequences of the strain have been uploaded to NCBI and the accession number is PV926768 (ITS), PV929713 (LSU), PZ097491(ACT).

## Appendix A

**Table A1:** Formulations of Culture Media and Reagents Used in the Experiment.

Culture Media	Drugs
Potato Dextrose Agar Medium (PDA)	Potato 200 g, Anhydrous dextrose 20 g, Agar 20 g, H_2_O 1000 mL
Potato Dextrose Medium (PDB)	Potato 200 g, Anhydrous dextrose 20 g, H_2_O 1000 mL
Czapek Medium	Sucrose 30 g, Agar 20 g, KCl 0.5 g, NaNO_3_ 2 g, K_2_HPO_4_ 1 g, FeSO_4_ 0.01 g, MgSO_4_·7H_2_O 0.5 g, H_2_O 1000 mL
Organic Phosphorus Medium	Anhydrous dextrose 10 g, Lecithin 5 g, Agar 20 g, (NH_4_)_2_SO_4_ 0.5 g, NaCl 0.3 g, K_2_SO_4_ 0.3 g, MgSO_4_·7H_2_O 0.3 g, MnSO_4_ 0.03 g, FeSO_4_ 0.03 g, H_2_O 1000 mL
Nitrogen-Free Medium	Mannitol 10 g, Agar 20 g, KH_2_PO_4_ 0.2 g, NaCl 0.2 g, CaSO_4_ 0.1 g, CaCO_3_ 5 g, MgSO_4_·7H_2_O 0.2 g, H_2_O 1000 mL
Siderophore Detection Medium	Sucrose 10 g, Peptone 5 g, Yeast Extract 1 g, Agar 20 g, KH_2_PO_4_ 0.2 g, MgSO_4_·7H_2_O 0.2 g, H_2_O 1000 mL
Aleksandrov Medium	Sucrose 5 g, Agar 20 g, Bromothymol Blue 0.1 g, Potassium Feldspar Powder 1 g, Na_2_HPO_4_ 2 g, MgSO_4_·7H_2_O 0.5 g, CaCO_3_ 0.1 g, FeCl_3_ 0.05 g, H_2_O 1000 mL
Amylase Detection Medium	Soluble Starch 1 g, Agar 2 g, K_2_HPO_4_ 0.03 g, KNO_3_ 0.1 g, NaCl 0.05 g, MgSO_4_·7H_2_O 0.1 g, H_2_O 1000 mL
Protease Detection Medium	Skim Milk Powder 5 g, Agar 20 g, Peptone 10 g, Beef Extract 3 g, NaCl 5 g, H_2_O 1000 mL
Cellulase Detection Medium	Peptone 5 g, Agar 20 g, Congo Red 0.05 g, CMC-Na 1 g, KH_2_PO_4_ 1 g, MgSO_4_·7H_2_O 0.5g, (NH_4_)_2_SO_4_ 1 g, H_2_O 1000 mL
*β*-Glucanase Detection Medium	Dextran 5 g, Congo Red 0.05 g, Agar 20 g, KCl 0.5 g, K_2_HPO_4_ 1 g, MgSO_4_·7H_2_O 0.5 g, NaNO_3_ 2 g, H_2_O 1000 mL
Pectinase Detection Medium	Pectin 10 g, Potassium Feldspar Powder 0.5 g, Anhydrous dextrose 1 g, Agar 20 g, Na_2_SO_4_ 1 g, KH_2_PO_4_ 1 g, KCl 1 g, MgSO_4_·7H_2_O 0.5 g, H_2_O 1000 mL
CAS assay solution	Chrome azurol S 60.5 mg, FeCl_3_·6H_2_O 2.645 mg (in 10 mL 10 mmol/L HCl), hexadecyltrimethylammonium bromide 72.9 mg, dissolved and made up to 100 mL with distilled water, filter-sterilized (0.22 μm).
Molybdenum-antimony anti-color reagent was prepared as follows	10.0 g of ammonium molybdate was dissolved in 200 mL of 5.0 mol/L sulfuric acid solution, then 0.3 g of antimony potassium tartrate was added and mixed thoroughly. The solution was diluted to 1 L with distilled water and stored in a brown bottle at 4 °C.
3,5-dinitrosalicylic acid (DNS)	3,5-dinitrosalicylic acid 6.3 g, NaOH 26.2 g, potassium sodium tartrate (Rochelle salt) 182.0 g, phenol 5.0 g, sodium sulfite 5.0 g, distilled water to 1000 mL. The reagent was stored in a brown bottle at 4 °C in the dark.

**Table A2:** Sequences used for phylogenetic tree construction.

Species Name	Strain Code	GenBank Accession Number		
		ITS	LSU	ACT
*Thelonectria veuillotiana*	GJS04-155	JQ403317.1	JQ403357.1	JQ365037.1
*Thelonectria amamiensis strain*	MAFF239820	JQ403338.1	JQ403376.1	JQ365055.1
*Thelonectria torulosa*	AR4764	JQ403309.1	JQ365030.1	JQ403349.1
*Thelonectria acrotyla*	GJS90-171	JQ403329.1	JQ403368.1	JQ365047.1
*Thelonectria gongylodes*	GJS89-131	JQ403336.1	JQ403374.1	JQ365053.1
*Thelonectria diademata*	AR4765	JQ403308.1	JQ403348.1	JQ365029.1
*Thelonectria coronata*	CTR71-79	JQ403315.1	JQ403355.1	JQ365035.1
*Thelonectria stemmata*	CTR71-21	JQ403313.1	JQ403353.1	JQ365034.1
*Thelonectria truncata*	GJS04-357	JQ403319.1	JQ403359.1	JQ365039.1
*Thelonectria olida culture*	CBS:215.67	MW827640.1	MH870642.1	HM352884.1
*Thelonectria truncata*	strain GJS 83-156	HM484559.1	HM364321.1	HM352887.1
*Thelonectria beijingensis*	HMAS 188498	JQ836656.1	MF669054.1	MF669047.1
*Thelonectria guangdongensis*	10627	MF669051.1	MF669053.1	MF669045.1
*Thelonectria porphyria*	5542	HM054136.1	HM042433.1	MK556798.1
*Thelonectria discophora*	GJS.10-118	KC153731.1	KC121457.1	KC121393.1
*Thelonectria japonica*	5621	HM054140.1	HM042434.1	MK556799.1
*Nectria cinnabarina*	4302	HM484548.1	HM484562.1	HM484627.1

## References

[1] Zhang X., Li S., Long Q., Yang Y. (2023). Identification of Phytopythium helicoides causing root rot of impatiens hawkeri and toxicity test of different fungicides in laboratory. Acta Hortic. Sin..

[2] Zhang D., Wei J., Zhou M., Li Y., Li X., Wen Y., Huang M., Huang H. (2022). Efficient plant regeneration system for New Guinea *Impatiens* (*Impatiens hawkeri W. Bull*) CV. ‘Violet’ and ‘Scarlet Bronze Leaf’. Plant Cell Tissue Organ Cult..

[3] Li H., Yang M., Liu J., Sun Y., Yang H., Lu J. (2025). Isolation and identification of antagonistic fungi for biocontrol of Impatiens hawkeri leaf spot disease and their growth-promoting potential. Front. Microbiol..

[4] Németh M.Z., Seress D., Nonomura T. (2023). Fungi Parasitizing Powdery Mildew Fungi: *Ampelomyces* Strains as Biocontrol Agents against Powdery Mildews. Agronomy.

[5] Li Q., Zhang D., Ye D., Zhang S., Ma Q., Bai H., Meng F. (2025). Biocontrol and Mycotoxin Mitigation: An Endophytic Fungus from Maize Exhibiting Dual Antagonism Against *Fusarium verticillioides* and Fumonisin Reduction. J. Fungi.

[6] Feng X., Jin Y., Zhong Z., Zheng Y., Wu H. (2025). Growth-Promoting Effects of Dark Septate Endophytes Fungus *Acrocalymma* on Tomato (*Solanum lycopersicum*). J. Fungi.

[7] Asad S., Priyashantha A.K.H., Tibpromma S., Luo Y., Zhang J., Fan Z., Zhao L., Shen K., Niu C., Lu L. (2023). Coffee-Associated Endophytes: Plant Growth Promotion and Crop Protection. Biology.

[8] Stakelienė V., Pašakinskienė I., Matijošiūtė S., Martūnas J., Štukėnienė G. (2025). Identifying Root-Associated Endophytic Fungi and Bacteria in *Festuca* and *Lolium* Grasses from a Site in Lithuania. Microorganisms.

[9] Valente I.d.L., Wancura J.H.C., Zabot G.L., Mazutti M.A. (2025). Endophytic and Rhizospheric Microorganisms: An Alternative for Sustainable, Organic, and Regenerative Bioinput Formulations for Modern Agriculture. Microorganisms.

[10] Kim M., Nguyen M.H., Lee S., Han W., Kim M., Jeon H., Lee J., Seo S., Kim N., Shin K. (2025). Diversity of Endophytic Fungi Isolated from *Prunus yedoensis* and Their Antifungal Activity Against Wood Decay Fungi. Microorganisms.

[11] Kharkwal A.C., Joshi H., Shandilya C., Dabral S., Kumar N., Varma A. (2024). Isolation and characterization of a newly discovered plant growth-promoting endophytic fungal strain from the genus *Talaromyces*. Sci. Rep..

[12] Franken P. (2012). The plant strengthening root endophyte *Piriformospora indica*: Potential application and the biology behind. Appl. Microbiol. Biotechnol..

[13] Galeano R.M.S., Silva S.M., Yonekawa M.K.A., de Alencar Guimarães N.C., Giannesi G.C., Masui D.C., Corrêa B.O., da Silva Brasil M., Zanoelo F.F. (2023). *Penicillium chrysogenum* strain 34-P promotes plant growth and improves initial development of maize under saline conditions. Rhizosphere.

[14] Xu D., Li N., Gu Y.Q., Huang J., Hu B.S., Zheng J.Y., Hu J.W., Du Q. (2023). Endophytic fungus *Colletotrichum* sp. AP12 promotes growth physiology and andrographolide biosynthesis in *Andrographis paniculata* (Burm. f.) Nees. Front. Plant Sci..

[15] Dai Z.-C., Fu W., Wan L.-Y., Cai H.-H., Wang N., Qi S.-S., Du D.-L. (2016). Different Growth Promoting Effects of Endophytic Bacteria on Invasive and Native Clonal Plants. Front. Plant Sci..

[16] Christakis C.A., Daskalogiannis G., Chatzaki A., Markakis E.A., Mermigka G., Sagia A., Rizzo G.F., Catara V., Lagkouvardos I., Studholme D.J. (2021). Endophytic Bacterial Isolates From Halophytes Demonstrate Phytopathogen Biocontrol and Plant Growth Promotion Under High Salinity. Front. Microbiol..

[17] Kulkarni G.B., Sanjeevkumar S., Kirankumar B., Santoshkumar M., Karegoudar T.B. (2013). Indole-3-acetic acid biosynthesis in *Fusarium delphinoides* strain GPK, a causal agent of Wilt in Chickpea. Appl. Biochem. Biotechnol..

[18] Gao Y., Ning Q., Yang Y., Liu Y., Niu S., Hu X., Pan H., Bu Z., Chen N., Guo J. (2021). Endophytic *Streptomyces hygroscopicus* OsiSh-2-Mediated Balancing between Growth and Disease Resistance in Host Rice. mBio.

[19] Baron N.C., Rigobelo E.C. (2022). *Endophytic fungi*: A tool for plant growth promotion and sustainable agriculture. Mycology.

[20] Adeleke B.S., Ayilara M.S., Akinola S.A., Babalola O.O. (2022). Biocontrol mechanisms of endophytic fungi. Egypt. J. Biol. Pest Control.

[21] Salgado J.F.M., Hervé V., Vera M.A.G., Tokuda G., Brune A. (2024). Unveiling lignocellulolytic potential: A genomic exploration of bacterial lineages within the termite gut. Microbiome.

[22] Ayob F.W., Simarani K. (2016). Endophytic filamentous fungi from a Catharanthus roseus: Identification and its hydrolytic enzymes. Saudi Pharm. J..

[23] Sharma S., Dhar M.K., Kaul S. (2023). Antagonistic, plant growth promoting and extracellular hydrolytic enzyme activity of fungal endophytes of *Dioscorea bulbifera* L.. Biocatal. Agric. Biotechnol..

[24] Kuo J., Chang C.F., Chi W.C. (2021). Isolation of endophytic fungi with antimicrobial activity from medicinal plant *Zanthoxylum simulans* Hance. Folia Microbiol..

[25] Putri N.D., Sulistyowati L., Aini L.Q., Muhibuddin A., Trianti I. (2022). Screening of endophytic fungi as potential antagonistic agents of *Pyricularia oryzae* and evaluation of their ability in producing hydrolytic enzymes. Biodiversitas J. Biol. Diver..

[26] dos Reis J.B.A., Lorenzi A.S., do Vale H.M.M. (2022). Methods used for the study of endophytic fungi: A review on methodologies and challenges, and associated tips. Arch. Microbiol..

[27] Claerbout J., Van Poucke K., Mestdagh H., Delaere I., Vandevelde I., Venneman S. (2023). *Fusarium isolates* from Belgium causing wilt in lettuce show genetic and pathogenic diversity. Plant Pathol..

[28] Esmaeili Taheri A., Chatterton S., Foroud N.A., Gossen B.D., McLaren D.L. (2017). Identification and community dynamics of fungi associated with root, crown, and foot rot of field pea in western Canada. Eur. J. Plant Pathol..

[29] O’Donnell K., Whitaker B.K., Laraba I., Proctor R.H., Brown D.W., Broders K., Kim H.S., McCormick S.P., Busman M., Aoki T. (2022). DNA Sequence-Based Identification of Fusarium: A Work in Progress. Plant Dis..

[30] Zhang X., Rajendran A., Grimm S., Sun X., Lin H., He R., Hu B. (2023). Screening of calcium- and iron-targeted phosphorus solubilizing fungi for agriculture production. Rhizosphere.

[31] Doilom M., Guo J.W., Phookamsak R., Mortimer P.E., Karunarathna S.C., Dong W., Liao C.F., Yan K., Pem D., Suwannarach N. (2020). Screening of Phosphate-Solubilizing Fungi From Air and Soil in Yunnan, China: Four Novel Species in *Aspergillus*, *Gongronella*, *Penicillium*, and *Talaromyces*. Front. Microbiol..

[32] Ozimek E., Hanaka A. (2021). *Mortierella* Species as the Plant Growth-Promoting Fungi Present in the Agricultural Soils. Agriculture.

[33] Yang S., Zhang X., Cao Z., Zhao K., Wang S., Chen M., Hu X. (2014). Growth-promoting *Sphingomonas paucimobilis* ZJSH1 associated with *Dendrobium officinale* through phytohormone production and nitrogen fixation. Microb. Biotechnol..

[34] Imran M., Abulreesh H.H., Monjed M.K., Elbanna K., Samreen, Ahmad I. (2021). Multifarious functional traits of free-living rhizospheric fungi, with special reference to *Aspergillus* spp. isolated from North Indian soil, and their inoculation effect on plant growth. Ann. Microbiol..

[35] Machado-Rosa T.A., Barbosa E.T., Cortes M.V.B.d.C., Lobo M. (2023). Microtiter method for quantitative assay of IAA from fungal isolates, demonstrated with Trichoderma. Rhizosphere.

[36] González Pereyra M.L., Di Giacomo A.L., Lara A.L., Martínez M.P., Cavaglieri L. (2020). Aflatoxin-degrading *Bacillus* sp. strains degrade zearalenone and produce proteases, amylases and cellulases of agro-industrial interest. Toxicon Off. J. Int. Soc. Toxinology.

[37] Zhou J., Xie Y., Liao Y., Li X., Li Y., Li S., Ma X., Lei S., Lin F., Jiang W. (2022). Characterization of a *Bacillus velezensis* strain isolated from *Bolbostemmatis Rhizoma* displaying strong antagonistic activities against a variety of rice pathogens. Front. Microbiol..

[38] Gorai P.S., Ghosh R., Mandal S., Ghosh S., Chatterjee S., Gond S.K., Mandal N.C. (2021). *Bacillus siamensis* CNE6- a multifaceted plant growth promoting endophyte of *Cicer arietinum* L. having broad spectrum antifungal activities and host colonizing potential. Microbiol. Res..

[39] Daigham G.E., Mahfouz A.Y., Abdelaziz A.M., Nofel M.M., Attia M.S. (2024). Protective role of plant growth-promoting fungi *Aspergillus chevalieri* OP593083 and *Aspergillus egyptiacus* OP593080 as biocontrol approach against *Alternaria* leaf spot disease of Vicia faba plant. Biomass Convers. Biorefinery.

[40] Parveen S., Wani A.H., Bhat M.Y. (2019). Effect of culture filtrates of pathogenic and antagonistic fungi on seed germination of some economically important vegetables. Braz. J. Biol. Sci..

[41] Taha A.S., Fathey H.A., Mohamed A.H., Ibrahim A.A., Abdel-Haleem M. (2025). Mitigating drought stress and enhancing maize resistance through biopriming with *Rhizopus arrhizus*: Insights into Morpho-Biochemical and molecular adjustments. BMC Plant Biol..

[42] Li J., Li J., Luo J., Li J., Sohail H., Zhu K., Yang P. (2025). Brassinosteroid-mediated carotenoid regulation enhances chilling tolerance in pepper. BMC Genom..

[43] Shen Z., Luo Q., Mao J., Li Y., Wang M., Zhang L. (2024). Molecular identification of two thioredoxin genes and their function in antioxidant defense in *Arma chinensis* diapause. Front. Physiol..

[44] Dulay R.M.R., Cabrera E.C., Kalaw S.P., Reyes R.G. (2021). Optimization of submerged culture conditions for mycelial biomass production of fourteen Lentinus isolates from Luzon Island, Philippines. Biocatal. Agric. Biotechnol..

[45] Das K., You Y.H., Lee S.Y., Jung H.Y. (2020). A New Species of Thelonectria and a New Record of *Cephalotrichum hinnuleum* from Gunwi and Ulleungdo in Korea. Mycobiology.

[46] Chaverri P., Salgado C., Hirooka Y., Rossman A.Y., Samuels G.J. (2011). Delimitation of *Neonectria* and *Cylindrocarpon* (*Nectriaceae*, *Hypocreales*, *Ascomycota*) and related genera with *Cylindrocarpon*-like anamorphs. Stud. Mycol..

[47] Zhuang W. (2016). Three new Chinese records of Nectriaceae. Mycosystema.

[48] Bong-Hyung L.B.L., DongYeo K.D.K., Hyeok Park P.H., Ahn-Heum E.A.E. (2016). Notes on Endophytic Fungi Isolated from Roots of *Oreorchis patens* in Korea. Korean J. Mycol..

[49] Muthu Narayanan M., Ahmad N., Shivanand P., Metali F. (2022). The Role of Endophytes in Combating Fungal- and Bacterial-Induced Stress in Plants. Molecules.

[50] Patel J., Teli B., Bajpai R., Meher J., Rashid M., Mukherjee A., Yadav S.K., Kumar A., Singh A.K., Choudhary K.K. (2019). 13—Trichoderma-mediated biocontrol and growth promotion in plants: An endophytic approach. Role of Plant Growth Promoting Microorganisms in Sustainable Agriculture and Nanotechnology.

[51] Ajuna H.B., Lim H.-I., Moon J.-H., Won S.-J., Choub V., Choi S.-I., Yun J.-Y., Ahn Y.S. (2023). The Prospect of Hydrolytic Enzymes from Bacillus Species in the Biological Control of Pests and Diseases in Forest and Fruit Tree Production. Int. J. Mol. Sci..

[52] Zhao X., Ding Z., Tao Z., Lyu D., Qin S. (2025). Isolation and Identification of plant growth promoting rhizobacteria from *Malus hupehensis* Rehd and Their Promoting Effects on the Horticultural Plants. Sci. Hortic..

[53] Zhou Y., Liu D., Li F., Dong Y., Jin Z., Liao Y., Li X., Peng S., Delgado-Baquerizo M., Li X. (2024). Superiority of native soil core microbiomes in supporting plant growth. Nat. Commun..

[54] El-Egami H.M., Hegab R.H., Montaser H., El-Hawary M.M., Hasanuzzaman M. (2024). Impact of Potassium-Solubilizing Microorganisms with Potassium Sources on the Growth, Physiology, and Productivity of Wheat Crop under Salt-Affected Soil Conditions. Agronomy.

[55] Zhou M., Li Y., Yao X.-L., Zhang J., Liu S., Cao H.-R., Bai S., Chen C.-Q., Zhang D.-X., Xu A. (2024). Inorganic nitrogen inhibits symbiotic nitrogen fixation through blocking NRAMP2-mediated iron delivery in soybean nodules. Nat. Commun..

[56] Gong W.K., Wu Q.H., Wang G., Yang J.M., Lu R.T., Wang B.B. (2024). Screening of microorganisms promoting rhizosphere growth in dragon fruit. J. Trop. Biol..

[57] Moropana T.J., Jansen Van Rensburg E.L., Makulana L., Phasha N.N. (2024). Screening *Aspergillus flavus*, *Talaromyces purpureogenus*, and *Trichoderma koningiopsis* for Plant-Growth-Promoting Traits: A Study on Phosphate Solubilization, IAA Production, and Siderophore Synthesis. J. Fungi.

[58] Mulatu A., Alemu T., Megersa N., Vetukuri R.R. (2021). Optimization of Culture Conditions and Production of Bio-Fungicides from *Trichoderma* Species under Solid-State Fermentation Using Mathematical Modeling. Microorganisms.

[59] Castro R.P., Dinho da Silva P., Pires L.C.C. (2024). Advances in Solutions to Improve the Energy Performance of Agricultural Greenhouses: A Comprehensive Review. Appl. Sci..

[60] Hannula S.E., Heinen R., Huberty M., Steinauer K., De Long J.R., Jongen R., Bezemer T.M. (2021). Persistence of plant-mediated microbial soil legacy effects in soil and inside roots. Nat. Commun..

